# Development of the equine gut microbiota

**DOI:** 10.1038/s41598-019-50563-9

**Published:** 2019-10-08

**Authors:** F. Lindenberg, L. Krych, W. Kot, J. Fielden, H. Frøkiær, G. van Galen, D. S. Nielsen, A. K. Hansen

**Affiliations:** 1 Brogaarden Aps, Copenhagen, Denmark; 2 Faculty of Health and Medical Sciences, Department of Veterinary and Animal Sciences, University of Copenhagen, Copenhagen, Denmark; 3 Faculty of Sciences, Department of Food Science, University of Copenhagen, Copenhagen, Denmark; 4 Department of Environmental Sciences, Aarhus University, København, Denmark; 5 Faculty of Health and Medical Sciences, Department of Veterinary Clinical Sciences, University of Copenhagen, Copenhagen, Denmark

**Keywords:** Mucosal immunology, Microbiome

## Abstract

Shortly after birth the mammalian gut is colonized, by a transient microbiota, highly susceptible to environment and diet, that eventually stabilizes and becomes the resident gut microbiota. In a window of opportunity during the colonization, oral tolerance is established towards resident bacteria. In this study, the development of the equine gut microbiota was investigated in ten foals from parturition until post weaning. We found great differences in the core species of the gut microbiota composition between time-matched samples on Day 7 and 20 post-partum. Between day 20 and Day 50 post-partum, we saw the gut microbiota became increasingly dominated by fiber fermenting species. After Day 50, no significant changes in species abundance were observed. Gene expression analysis of pro- and anti-inflammatory cytokines in the blood revealed no significant changes before and after weaning. In summary, relative stability of the gut microbiota was reached within 50 days post-partum and, weaning did not have a major impact on the microbial composition.

## Introduction

The mammalian gut is colonized by microorganisms immediately after birth, and the composition of the gut microbiota influences the development of the immune system^1,2^. An immunological homeostasis is established with oral tolerance toward food antigens and commensal bacteria^3^. Improper colonization and pathological changes in the microbial composition, called dysbiosis, upsets the homeostasis and often result in inflammation and have been shown to govern inflammatory and metabolic diseases in animal models^4–8^. Postnatal acquirement of a health- promoting gut microbiota is, therefore, a key factor in ensuring the establishment of a healthy immunological homeostasis. Dietary manipulation of the developing gut microbiota may promote the growth of beneficial bacterial species and has been demonstrated to influence the immune system in humans and mice positively^9,10^. To secure that a dietary intervention has a beneficial and lasting impact on the microbiota and immune system in foals, it is necessary to identify the time point at which the microbiota stabilizes.

Initial colonization, stabilization of the microbiota, weaning, and transfer to solid food are major events responsible for shaping of the gut microbiota and immunological homeostasis in mammals^11^. Several studies have pinpointed conversion to solid food and weaning as the time at which the mature gut microbiota is permanently established, and there is substantial evidence that this period is also vital in shaping the immune system^12,13^. The importance of the pre-weaning period is shown by studies observing significant differences in microbial composition, expression of immune receptors, cytokines and signaling pathways in germ-free mice colonized before and after three weeks of age^1,14^. This indicates the existence of a window in which the immune system is susceptible to gut microbiota modulations which alter the immunological phenotype permanently^15^.

Weaning of foals usually takes place between 4 to 6 months of age, but the change from milk to solid foods is gradual, as most foals will have started ingesting solid food before weaning^16^. Culture techniques identifying selected species reported high similarity between foal and mare microbiota four weeks after parturition^17^. Denaturation Gradient Gel Electrophoresis (DGGE) band patterns between mares and foals were similar 42 days after birth^18^. Automated Ribosomal Intergenic Spacer Analysis (ARISA) revealed no shifts in community structure after 30 days of age^19^. 16S ribosomal ribonucleic acid (rRNA) sequencing revealed relative stability after 60 days of life, with minor differences still evident between nine-month-old foals and their mares^20^. The effect of weaning was investigated with both fingerprinting and sequencing, and both studies showed little or no effect on the composition^19,20^, which is incongruent with findings in other species, in which considerable changes in the gut microbiota have been attributed to weaning as described above. Based on the inter- study differences a conclusion regarding the time point for microbiota stabilization and the effect of weaning cannot be reached. However, major changes in microbial composition seem to occur within the first couple of months after birth, before weaning.

The aim of this study was to use next generations sequencing to determine the period of gut microbiota stabilization in foals. Considering the importance of weaning in the development of the gut microbiota and the immune system in other species, we also wished to assess the possible changes in the microbial composition and inflammatory markers before and after weaning.

## Method

### Animals

All assays were carried out on samples collected as part of the stud farm’s routine health surveillance, by an authorized veterinarian, which according to the Animal Experimentation Act (LBK No. 474 15/05/2014, should not be regarded as animal experimentations. All procedures were carried out according to The Danish Veterinary Act (Dyrlægeloven) no 48 of11/01/2017. Approval was obtained from the owners prior to the study start.

Ten mares with foals housed at a privately owned stud farm were included (York stud farm, Hørsholm, Denmark). All mares were Thoroughbreds that were housed under high-level hygienic conditions and clinically healthy based on history and clinical examination. The foals were born in the period from February to April 2015. Gestational lengths (334 to 356 days) and deliveries were normal. Only one mare needed veterinary assistance during parturition, and this mare received post-partum antibiotics (penicillin/gentamycin (Genta-equine, Dechra) one dose intra muscularly, prophylactically). All foals stood within 2 hours and nursed within 6 hours and none of them developed signs of disease at any point. Serum immunoglobulin G (IgG) was systemically measured 12 hours after birth in a semi-quantitative enzyme-linked immunosorbent assay (ELISA) quick-test (KRUUSE IgG Foal Quick Test, KRUUSE, Langeskov, Denmark). Foals with IgG levels under 400 mg/dl received a colostrum replacement pasta (FoalGard, Hunden och Herden, Sjöbo, Sweden) once within 24 hours post-partum. All but two foals had IgG levels above 400 mg/dl. No other replacement, bottle- or tube feeding was given. Seven of the foals developed mild, self-limiting “foal heat diarrhea” around the age of two weeks post-partum. No “foal heat diarrhea” exceeded the time between aged 7–12 days. Mares were housed with their foals in individual boxes (one box per mare/foal pair) and let out in a pen in foal/mare pairs but separately from other pairs for the first month. New straw was added to the boxes daily, and boxes were mocked out once a week. Mares and foals were turned out on pasture daily in small groups when the foals were approximately one month old. They remained full time on pasture from June until weaning.

Foals were not fed separately but had access to the mares feed bucket throughout the study period. The mares were fed a concentrate for mares and growing foals (Optimal no. 1 Suregrow, Brogaarden Diets Ltd. Lynge, Denmark) (Supplemental Table 1). Pre partum and for the first two months post-partum mares received 1–1.5 kg/day, from one month post-partum doses were gradually increased to 1.5–2.3 kg/day as foals started ingesting more concentrate. Additionally, mares were fed lucerne (0.75–1 kg/day) and hay ad libitum. Free access to clean water was available at all times for foals and mares.

All foals were weaned simultaneously regardless of age at the end of November. Weaning took place over a week when foals were gradually weaned from the mare and introduced to co-housing in a barn.

### Collection of samples

Parturition was considered as Day 0, and fecal samples were collected from foals on Day 7, 20, 50, 80, 110, and 140 post-partum. Additionally, a jugular blood sample and a fecal sample was collected immediately pre- (age 262+/−22 days) and approximately 14 days post weaning (age 280+/−18 days). A fecal sample was taken from all mares immediately pre weaning to be compared to that of the foals. All samples were collected as fecal grab samples immediately after leaving the rectum. When possible a subsample of about 100–200 grams was collected from the part of the feces that had not been into contact with the ground, when not the entire defecations was collected. From June foals and mares were let out on permanent pasture, which made sampling significantly more difficult, and samples collected between Day 80 and 110 were combined into one time point, because successful sampling did not happen in all cases. No samples were taken from foals suffering from the foal heat diarrhea (5 missing samples on day 7).

All fecal samples were collected on site into 50 mL Falcon Tubes (Thermo Fischer Scientific Denmark) and stored on −20 °C in a mobile freezer (max 3 hours) until it could be stored in a permanent freezer facility at −80 °C.

For quantitative polymerase chain reaction (qPCR) gene expression analysis, blood was collected from the jugular vein and processed as described by Mærkedal *et al*.^21^. Briefly, samples were collected from the jugular vein by a Vacutainer^TM^ (Kruuse, Denmark) needle into 6 mL EDTA coated vacuum tubes (BD, Kruuse, Langeskov, DK) from which 0.5 mL whole blood was transferred to Eppendorf tubes containing 1.3 mL MagMax Lysis/Binding solution concentrate (Ambion, Thermo Fischer Scientific Denmark) and stored at −80 °C until processing.

### Fecal samples analysis

#### Extraction of DNA

Bacterial DNA (Deoxyribonucleic acid) was extracted using the MoBio Power Soil Kit (MoBio QIAGEN Nordic, Copenhagen East). A pre step was included were samples were thawed, and 20 grams were suspended in 40 ml sterile milli-Q water in a stomacher bag with filter and processed in a stomacher (Seward 80 BA 7020 Stomacher Lab Blender, Seward, West Sussex, United Kingdom) in 2 min at maximum speed. A volume of 200 μl was then used for DNA-extraction. The DNA extraction was performed according to the manual.

#### High throughput sequencing of the gut microbiota

Fecal microbiota composition was determined using tag-encoded 16S rRNA gene (V3-V4-region) MiSeq-based (Illumina, San Diego, CA) high throughput sequencing. DNA extraction, storage conditions, and sequencing library preparation were conducted as previously described^22^.

### Sequencing data analysis

The raw dataset containing pair-ended reads and quality scores were merged, trimmed according to quality scores, clustered to operational taxonomic units (OTUs), and corrected for chimeras using UPARSE pipeline^23^ using setting as previously described^22^. The green genes (13.8) 16 S rRNA gene collection was used as a reference database.

Not all foals were represented at all times points due to the difficulty of obtaining samples from young foals. To achieve a higher group size and better statistical robustness in the analysis samples were grouped as follows: Day 7 and Day 20 (time 1), Day 50 and Day 80–110 (time 2), and Day 140 and pre-weaning (time 3). Samples taken post weaning were not grouped with others in order to enable assessment of pre- and post-weaning effects on the microbial composition.

### Gene expression analysis

Ribonucleic acid (RNA) was extracted using the MagMAX-96 Blood RNA Isolation kit (Ambion, Fischer Scientific, Denmark) and the MagMAX Express Magnetic Particle Processor (Fischer Scientific Denmark). Procedures were carried out according to the manufacturers manual with slight adjustments as described by Mærkedahl *et al*.^21^. RNA concentration and purity was measured on Nanodrop 2000 (Thermo Fischer). Samples (200 ng of each sample) were aliquoted in a polymerase chain reaction (PCR) -plate and the Applied Biosystem High-Capacity cDNA (Complimentary deoxyribonucleic acid) Reverse Transcriptase Kit (Applied Biosystems, Fischer Scientific, Denmark) with random primers and a total volume of 10 μl was used for cDNA synthesis. Procedures were carried out according to the manufacturer’s protocol. qPCR was performed using the TaqMan Fast Universal PCR Master Mix (Applied Biosystems, Fischer Scientific Denmark) on 2 μl cDNA product and a total volume of 10 μl. Equine probes were obtained from Applied Biosystems, Thermo Fischer Scientific and analyses were carried out in duplicates for each samples for interleukin 6 (*il6*) (probe ID: Ec03468680_m1), interleukin 10 (*il10*) (probe ID: Ec03468647_m1), interleukin 1 beta (*il1b*) (probe ID: Ec_04260289_s1), transforming growth factor beta (*tgfb*) (probe ID: Ec03468030_m1), and 18 s ribosomal ribonucleic acid (*18 s)* (probe ID: 4333760 F) on the StepOnePlus instrument (Applied Biosystems, Fischer Scientific Denmark). The amplification data was analyzed using the StepOne v 2.3 software (Applied Biosystems, Fischer Scientific Denmark) to obtain threshold cycle (C_T_) values. Quality check of the amplification data was performed and samples flagged NOSIGNAL (No detectable level of fluorescence), NOAMP (No amplification), NOISE (The background fluorescence in the well exceeds the limit) were excluded from the analysis.

### Statistical analysis

Quantitative Insight Into Microbial Ecology (QIIME) open source software packages (1.7.0, 1.8.0 and 1.9.1)^24,25^ were used for analysis of the sequencing data. Alpha diversity expressed with an observed species (sequence similarity 97% OTUs), Chao1 and Shannon indices were computed based on rarefied OTU tables (10,000 reads/sample) using the alpha rarefaction workflow (QIIME V1.9). Differences in alpha diversity were determined using a t-test-based approach employing the non-parametric (Monte Carlo) method (999 permutations) implemented in the compare alpha diversity workflow. Unweighted and weighted UniFrac distance matrices were calculated from subsampled OTU tables (10.000 reads/sample) and visualized with Principal Coordinates Analysis (PCoA) plots. The separation between the time groups was tested with Permutational Multivariate Analysis of Variance (PERMANOVA). Comparison of UniFrac distances between given categories was performed using “make_distance_poxplot” workflow (QIIME 1.8.0) performing two-sample t-tests between time points. The differences in taxa abundance between categories were estimated with a statistic framework: analysis of composition of microbes (ANCOM) based on raw OTU-table^26^. Graphpad Prism (GraphPad Software, Inc. La Jolla, CA, USA) was used to generate the alpha-diversity plots and analyze qPCR data. C_T_ values were obtained from the qPCR and fold changes were calculated according to the delta-delta-Threshold-Cycle ddCt (delta delta threshold cycle) method^27^. The average expression of the target genes as normalized to the reference gene 18S [dC_T_(Delta threshold cycle)_sample)_ = C_T(target)_ − C_T(reference)_]. The average gene expression level measured before weaning was used as calibrator and gene expression was calculated as C_T(sample)_ − C_T(calibrator)_. Normal distribution was assessed by a Shapiro-Wilk normality test and data that did not follow a normal distribution was analyzed by a non-parametric Mann-Whitney test. P values below 0.05 were considered significant.

## Results

**Figure 1:**
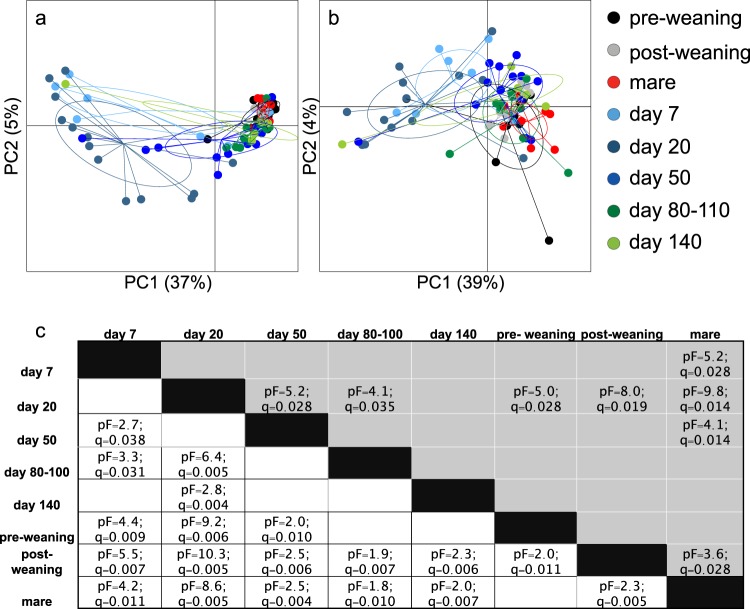
PCoA plots based on unweighted (**a**), and weighted (**b**) UniFrac distance matrices reflecting similarities in microbial communities between fecal samples of foals collected from birth until after weaning and one time matched samples from mares taken together with the “before weaning” samples. Analysis shows clear qualitative (**a**), and quantitative (**b**) differences in microbial communities according to time. Paired PERMANOVA results for weighted (grey area) and unweighted (white area) UniFrac distance matrices are given in table (**c**).

PCoA plots based on unweighted and weighted UniFrac distances show significant differences between the samples collected at the different time points (Fig. 1). Samples collected at Day 7 and Day 20 clustered together and presented more separation both unweighted (Fig. 1a) and weighted (Fig. 1b). Paired PERMANOVA results showed a tendency towards less and less separation with increased age up until just before weaning, most notably in the analysis in unweighted UniFrac distances (Fig. 1c). UniFrac distances for samples taken pre- and post weaning showed significant separation with all other times and between pre- and post weaning. No differences were observed between pre-weaning samples collected from foals and mares, but between mares and samples collected post weaning.

**Figure 2:**
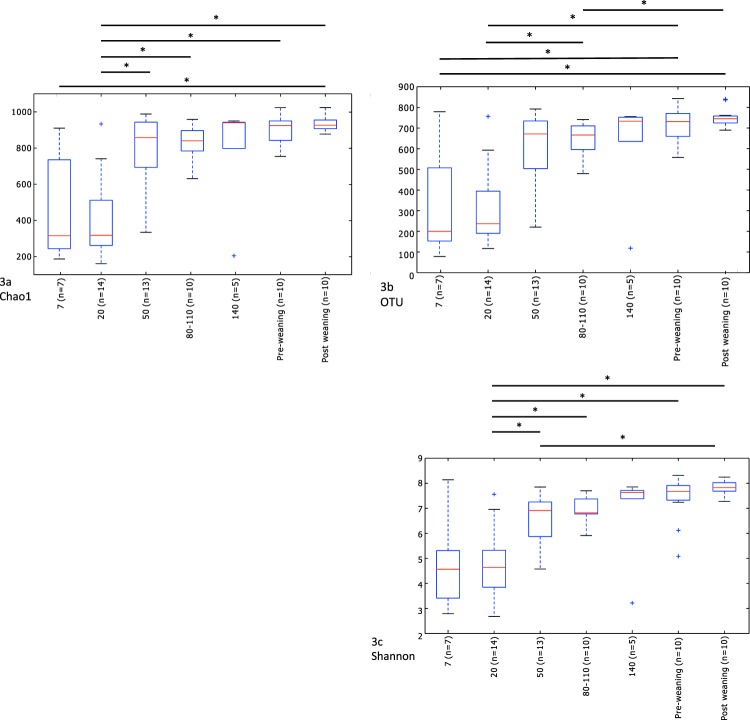
Alpha diversity shown as Chao1 (**a**), Observed species (**b**) and on the Shannon index (**c**) (mean, SD) calculated based on rarefied (10.000 reads/sample) OTU-table for foals on Day 7, 20, 50, 80–110, 140 post-partum, pre- and 14 days post weaning. Significant differences (p < 0.05) are indicated wit a*.

From parturition to post weaning there was a steady increase in alpha diversity determined as Chao1, observed species and as the Shannon Index (Fig. 2a–c and Supplementary Table S2). There was a steady increase in alpha diversity over time on all indices. Most notably between day 20 and 50. No significant differences were observed between samples taken pre- and post-weaning.

**Figure 3:**
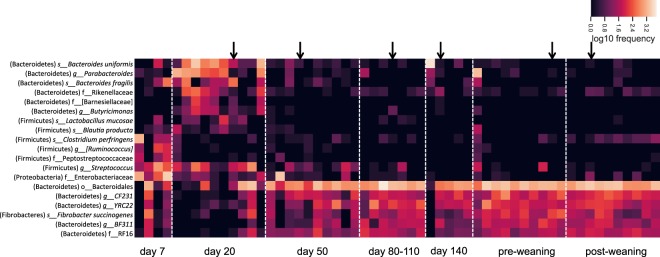
Heatmap illustrating taxa characterized with amplicon sequencing of 16S rRNA gene (v3-v4 region), which differed significantly in abundances (ANCOM, p < 0.05) from fecal samples taken from foals at different times. Only major differences in taxa abundance were between 20 weeks and the rest. Scale on the right indicates log abundance. Arrow indicate foal from mare who received post-partum antibiotics.

**Figure 4:**
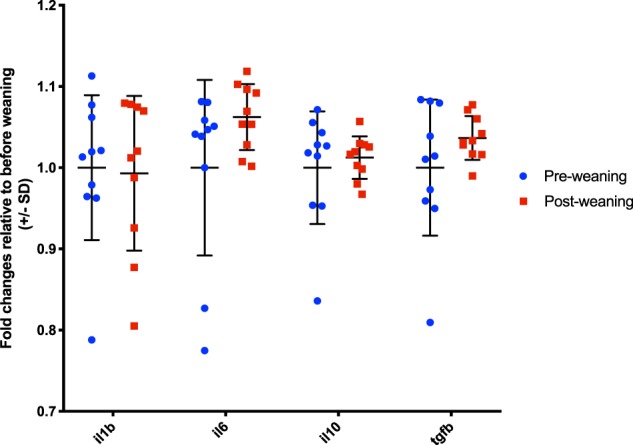
Plot showing gene expression of immunological parameters before and after weaning measured by qPCR on blood RNA levels and calibrated to the average gene expression before weaning. No significant differences were found in gene expression before and after weaning.

ANCOM analysis revealed significant differences in composition between samples taken at Day 20 and samples taken at Day 50 and onwards (Day 80–110, Day 140, pre-weaning, post-weaning, mares) (Fig. 3). Before Day 50, the abundances of *BF311*, *CF231*, *YRC22*, *Rikenellaceae and RF16* (all Bacteroidetes) were significantly (q < 0.05) lower (Fig. 4). After Day 50, significant (q < 0.05) increases were observed in abundances of *Bacteroides uniformis*, *Bacteroides fragilis*, *Parabacteroides*, and *Butyricimonas* (all Bacteroidetes), Enterobacteriaceae (Proteobacteria) and, *Lactobacillus mucosae*, *Blautia producta*, and *Streptococcus* (all Firmicutes) (Fig. 3). Samples taken from the foal of the mare who received post-partum antibiotics followed the clustering patterns of the other samples in the respective time group (the samples have been marked in Fig. 3)

Analysis of gene expression showed no differences in any of the immune parameters measured pre and post weaning (Fig. 4).

## Discussion

This study demonstrated a significantly higher diversity in gut microbiota between time-matched samples taken on Day 7 and Day 20 compared to samples taken at day 50 and until after weaning, which indicates instabilities in the gut microbiota composition in the early period. This is very similar to the colonization patterns observed in other species in which the initial microbiota is very dynamic and characterized by transient colonization of bacteria that disappear after a short while^11^. In humans, the initial microbiota is acquired largely from the mother during birth, from suckling, and from the milk^28^. The results show a large inter -individual gut microbiota composition of foals at 7 days of age, indicating that at this early stage, the gut microbiota is very transient. At day 20 we see a more uniform microbiota composition between the foals, but a microbiota composition, that significantly differs from that of foals aged 50 days and older. Of the microbiota found in foals aged 20 days, *Lactobacillus mucosae* have been found in the vagina of mares^29^ and Streptococcus in the amniotic fluid^30^ suggesting that the foals might have gained part of their microbiota from the birth canal. However, both *Lactobacillus* and *Streptococci* have also previously been found in high numbers in mare’s milk^30^,^31^ and in human milk along with *Bacteroides*, and *Blautia*^32^. This could suggest that milk has a strong impact on the microbial composition in this period. Qercia *et al*.^30^ investigated the vertical transfer of microbiota from mares to foals, and found that the milk and foal gut microbiota shares similarities early on, but also that coprophagia played a large part in shaping the early gut microbiota. The coprophagic behavior shown in foals almost immediately after birth may contribute to stabilization by providing a steady source of microbiota early on. Coprophagic behavior has been observed to be most pronounced during the first two months of life^33^. This approximately coincides with the time in which we observe the majority of the microbial establishment, suggesting the vertical transfer of microbiota plays a role in microbial stabilization.

In addition to directly serving as a source of microorganisms via the glands, the mare’s milk is also rich in oligosaccharides, which play a major role in shaping the microbiota^34^. Changes in both quality and quantity of milk oligosaccharides have been shown to affect the microbial composition in pigs^35^, and oligosaccharides from the milk may act as a stabilizing agent on the microbiota early in life.

Like Bordin *et al*.^36^ who observed a higher abundance of Bacteroides in 30- day old foals, we found a significant increase of Bacteroides after day 50. Bordin *et al*. speculated that, as in humans, the gut microbiota gradually develops to consist of more and more anaerobic bacteria, which reflects the conditions of the adult GI tract. We have previously identified both *CF231*, *BF311, and RF16* (all Bacteroidetes) as well as *Fibrobacteres succinogenes* (Fibrobacter) as belonging to the core microbiome of adult horses^37^ and the significant increase in these species observed after Day 50 suggests that the adult microbiota is partly present already at this time. Additionally, foals start eating the mares’ food, hay and grass already when they are a couple of weeks old. A fiber-rich diet has been shown to promote the growth of fiber-fermenting bacteria such as *CF231 and BF311*^38^. De La Torre *et al*.^39^ found an increase in fiber fermenting bacteria from age 7 days supporting the assumptions that both coprophagia and the diet influence the gut microbial composition, even though the foals are still suckling.

A general increase in species richness (alpha-diversity) was observed until post- weaning, with a large increase in samples taken at Day 50 compared to samples taken at Day 7 and 20. This has been reported by other studies as well, who also found significant increases earlier than 50 days^40^. An increase in alpha-diversity and approximation to the mother’s composition has been associated with maturation of the gut microbiota in both humans and mice^41,42^. This further supports our assumption of stabilization occurring around Day 50.

As foals at weaning have had a subtle intake of hay, grass and mares food for at least several months, a microbial composition associated with this diet is to be expected. On the other hand, the cease in intake of mother’s milk could give rise to further changes due to the decreasing effects of specific milk components on the microbiota. The changes in microbial composition and immune response associated with weaning in both humans and mice^1,43^ prompted us to look into the possible compositional and immunological changes around weaning in horses. The beta-diversity showed significantly less separation in foals post- weaning compared to pre- weaning, indicating gut microbial changes. No taxa were found to be significantly different between pre- and post- weaning. Studies investigating the foal gut microbiota at weaning are still few, and results are not completely congruous. Costa *et al*.^20^, and De La Torre *et al*.^39^. Mach *et al*.^44^ observed changes in several more species than in the present study, when investigating two different types of weaning^45^. The studies differ in sampling times, feeding regiments and time and method of separation from the mare, making direct comparisons challenging. In the study by Mach^44^ the cereal diet was not introduced until one month before weaning, whereas foals in this study had access to cereal from birth, which may have promoted the maturation of the gut microbiota. Even though other studies have shown very little or none effect at all^19^, we hypothesize that milk may still have a minor effect on the gut microbiota composition. We reach this hypothesis as no other factors were changed at weaning. The diet remained the same and the foals continued being in the same fields as before weaning.

In mice changes in gut microbiota around the time of weaning affect the immune system and inflammatory level and result in differences in lymphocyte distribution and types^1,46^. Therefore, we wished to assess the immunological effects of weaning and especially the inflammatory responses to the suspected microbial changes pre- and post-weaning. Ideally, such changes should be assessed locally in the intestinal mucosa; however, it was considered too invasive to obtain biopsies of the mucosa. Hence, a panel of genes to analyze in blood extracted pre- and post-weaning was chosen to reflect the systemic status of the adaptive immune system. Several studies have shown that cytokines and chemokines are very unstable in unprocessed blood when stored in varying temperatures for longer times^47^. As the blood samples were collected in the stable and stored for up to a couple of hour before transport to the lab, gene expression analysis was chosen rather than protein measurement, as this was considered a more stable parameter. *il6* and *il1b* were chosen as markers of pro-inflammatory response and *il10*, and *tgfb* as markers of regulatory immunity. No differences in gene expression were detected between samples taken pre- and post-weaning, suggesting that no major immunological reactions or adaptations took place. This may indicate that milk consumption at the time of weaning does not have a major influence on the immune system. Other studies have found increased levels, and increased regulatory potential of forkhead box P3 (Foxp3) positive T-cells in foals during the first 3 months compared to older foals^48,49^, indicating that the immune system is better suited at establishing oral tolerance at this time. This corresponds with the period in which we observed the most prominent microbial stabilization and supports our hypothesis that this may be the most optimal time to influence the immune system through microbiota manipulation via dietary interventions.

The time of stabilization of the gut microbiota is not just interesting in relation to potential dietary interventions. Foal heat diarrhea has been associated with changes in gut microbiota^50^ suggesting that foal diarrhea may be susceptible to dietary interventions impacting the gut microbiota and be linked to the period before stabilization. In addition, foals are routinely treated with antibiotics. An antimicrobial treatment that removes or changes the abundance of certain species during the establishment of the immune-microbial homeostasis may have implications later in life as observed in mice and humans^51^.

The fact that all horses were housed at the same farm and thus have been exposed to many of the same environmental stimuli may have contributed to a more uniform microbial composition. Moving forward, multi-site studies should be conducted with enough animals to allow housing to be taken into account in the analysis. A limitation to the study is the relatively few samples in the Day 7 group and the only partial clustering with the Day 20 group. Bigger studies should be conducted with more foals in each group to gain a more robust statistical analysis.

## Conclusion

In conclusion, we observed marked stabilization of gut microbiota already around Day 50 post-partum. Weaning had some effects on the microbial stability but did not seem to be the cause of major immunological adaptations. This is probably due to the gradual introduction of adult diet long before weaning. The early microbiota was characterized by bacteria previously identified in milk and from the birth canal, while a significant increase in certain fiber fermenting species emerged from Day 50. The time window in which we observed the microbiota to stabilize corresponded with the time in which other studies have found high levels of reactive regulatory T-cells and anti-inflammatory cytokines in the blood. We, therefore, point at the period between birth and 50 days of age as the most likely window of opportunity for permanently influencing the immune system via the gut microbiota in horses.

## Supplementary information

Supplementary information

## Data Availability

Data and associated protocols are stored on the University of Copenhagen’s backup servers, and can be made available for other non-commercial research groups along with any remaining study materials, as far as still in stock, by communication with the corresponding author.
