# Supplementary material for: Development of the equine gut microbiota
Source: Sci Rep. 2019 Oct 8;9:14427. doi: 10.1038/s41598-019-50563-9 (PMC6783416; doi:10.1038/s41598-019-50563-9)
Supplement: Supplementary file 1 — Supplementary information [file 41598_2019_50563_MOESM1_ESM.pdf]

Development of the equine gut microbiota

F. Lindenberg[1,2*], L. Krych[3], W. Kot[4], J. Fielden[1], H. Frøkiær[2], G. van Galen[5], D. S. Nielsen[3], A. K. Hansen[2]

[1] Brogaarden Aps

[2] University of Copenhagen, Faculty of Health and Medical Sciences, Department of Veterinary and Animal Sciences

[3] University of Copenhagen, Faculty of Sciences, Department of Food Science

[4] Department of Environmental Sciences, Aarhus University

[5] University of Copenhagen, Faculty of Health and Medical Sciences, Department of Veterinary Clinical Sciences

[*]Correspondance to frederikke@lindenberg.dk

Diet

Analytical Constituents:

Crude Protein	0,25
Crude Oil and fats	5.4%
Crude Fiber	8.5%
Crude Ash	0,12
Sodium	0.5%

Digestible Energy MJ/kg: 11.75

Calcium	2.5%	Vitamin K	6mg/kg
Phosphorus	1.5%	Biotin	4.25mg/kg
Magnesium	0.4%	Pantothenic acid	65mg/kg
Salt	1.4%	Nicotinic acid	90mg/kg
Sodium	0.5%	Folic acid	80mg/kg
Potassium	1.2%	Choline Chloride	2000mg/kg
Sulphur	0.23%	Cobalt	1.1mg/kg
Lysine	18g/kg	Copper	165mg/kg

Methionine	3.7g/kg	Iron	250mg/kg
Vitamin A	54000Iu/kg	Manganese	350mg/kg
Vitamin D_3	5400iu/kg	Selenium	1.9mg/kg
Vitamin E	1200mg/kg	Zinc	550mg/kg
Vitamin B_1	62mg/kg	Iodine	2.2mg/kg
Vitamin B_2	25mg/kg	Starch	12-14%
Vitamin B_6	44mg/kg	Sugar	5-6%
Vitamin B_{12}	0.09mg/kg	DCAB	315mequiv
NDF	0,22	ADF	0,1

Supplemental table S1: Dietary composition

Day after birth	Chao1 Ave.	Chao1 Err	Observed species Ave.	Observed species Err.	Shannon Ave.	Shannon Err.
7	479.006	283.717	339.871	245.763	4.679	1.673
20	410.591	215.125	311.600	177.459	4.751	1.365
50	763.491	213.248	587.915	181.308	6.630	0,672
80-110	822.860	89.554	650.210	78.280	6.906	0,398
140	756.140	286.952	601.720	244.280	6.734	1.777
Before weaning	891.755	79.942	712.990	81.020	7.346	0,655
After weaning	938.367	46.066	760.020	46.379	7.821	0,196

Supplementary Table S2
Alpha diversity metrics for Chao1, Observed species and Shannon (average and error) calculated based on rarefied (10.000 reads/sample) OTU-table for foals on Day 7, 20, 50, 80-110, 140 post-partum, pre- and 14 days post weaning.
